# Impact of model physiotherapy centres in reducing the burden among the caregivers of children with neurodevelopmental disorders in the rural area of Tamil Nadu: a study protocol

**DOI:** 10.1186/s41043-024-00686-1

**Published:** 2024-12-18

**Authors:** Vadivelan Kanniappan, Prakash Muthuperumal, P. Venkataraman, T. S. Veeragoudhaman, Balaji Chinnasami, Manikumar Muthiah, Abishek Jayapal Rajeswari, Manju Bashini Manoharan, Shrisruthi Suresh, Ashok Natesan

**Affiliations:** 1 SRM College of Physiotherapy, Faculty of Medicine and Health Sciences, SRM Institute of Science and Technology, Kattankulathur, Chengalpattu, Tamil Nadu, 603203 India; 2 School of Public Health, Faculty of Medicine and Health Sciences, SRM Institute of Science and Technology, Kattankulathur, Chengalpattu, Tamil Nadu, 603203 India; 3 Department of Medical Research, Faculty of Medicine and Health Sciences, SRM Medical College Hospital and Research Centre, SRM Institute of Science and Technology, Kattankulathur, Chengalpattu, Tamil Nadu, 603203 India; 4 Department of Pediatrics, Faculty of Medicine and Health Sciences, SRM Medical College Hospital and Research Centre, SRM Institute of Science and Technology, Kattankulathur, Chengalpattu, Tamil Nadu, 603203 India

**Keywords:** Inequitable health access, Unnat Bharat Abhiyan, Model Clinic, Tamil Nadu, Physiotherapy, Neurodevelopment

## Abstract

**Background:**

Approximately 1 in 8 children under the age of 6 in Tamil Nadu are diagnosed with neurodevelopmental disorders (NDD), yet only a fraction of these children receives proper medical care. The unequal distribution of healthcare services is mainly due to the lack of accessibility, especially in rural areas, leading to a significant burden on caregivers. This research aims to alleviate caregiver burden and reduce disability in preschool children by establishing model clinics or specialized outreach centers in rural villages, supported by SRM Institute of Science and Technology under the Unnat Bharat Abhiyan Scheme.(UBA-SRMIST).

**Methods:**

The study will commence with screening all preschool children registered in Anganwadi in the designated villages. Tailored model clinics will be set up in these villages based on the prevalence of NDD. Once established, children diagnosed with NDD will undergo rehabilitation for a year. Baseline and endline assessments will be conducted to evaluate the effectiveness of the model center on both the child's disability and the caregiver burden.

**Discussion:**

This research will demonstrate the effectiveness of a model outreach center in rural villages in reducing disability levels in children and alleviating caregiver burden by eliminating the need for long travels to access rehabilitation services. The findings of this study will not only contribute to the objectives of UBA-SRMIST in uplifting villages but also facilitate the creation of registries and provision of data to the government for the implementation of policies that address the current disparities in healthcare access.

*Trial registration* Trail has been registered under Clinical Trials Registry—India (CTRI/2024/06/069196).

**Supplementary Information:**

The online version contains supplementary material available at 10.1186/s41043-024-00686-1.

## Background

Neurodevelopmental disorder (NDD) is a comprehensive term that encompasses a variety of conditions or dysfunctions that impede the growth and/or development of the nervous system, including issues such as visual impairments, specific learning disorders, autism, global developmental delay, Attention Deficit Hyperactivity Disorder(ADHD), Developmental Coordination Disorder (DCD) collectively impacting 13% of pediatric health cases. [1, 2].

Globally, more than 50 million children are recognized as having neurodevelopmental disabilities, with an estimated 23 million Indian children falling into this category, of whom only a small percentage receive medical attention.(2) The increasing prevalence suggests that one in eight Indian children experiences some form of NDD. In Tamil Nadu, it is approximated that 2% of children below the age of 6 have a form of disability, with many of them lacking medical care. [1–4].

The preschool age (3–5 years), which marks a critical period for functional brain development, neuronal migration, and cognitive and behavioral growth, and this group of children remains relatively unexplored. Despite the higher rate and risk of NDD development during this period, there is a lack of focus on it compared to infancy. This could be due to the challenges in screening or imaging assessments, resulting in a shortage of early detection methods. [5].

The alarming prevalence and long-term complications associated with NDD raise concerns regarding the need for specialized care, yet there is a shortage in the implementation of such care. This shortage is attributed to the limited access to specialized healthcare services. Various reasons contribute to this lack of access, including geographical barriers, economic constraints, cultural factors, lack of awareness, inadequate healthcare infrastructure, and insufficient healthcare workforce. [6, 7].

Data from National Family Health Survey (NFHS) II indicates that seven out of ten individuals in India lack access to healthcare, with even higher rates in rural areas as specialists are predominantly located in urban centers. Only one out of ten individuals have access to hospitals, and 13% have access to primary healthcare facilities. [7, 8].

The inequitable access to healthcare further compounds the situation, particularly in rural and economically disadvantaged settings in southern India, increasing the load on caregivers of children with NDD. As caregivers assume a pivotal role in the rehabilitation of children with NDD, their heightened responsibilities/burden directly influence the caliber of care extended to the child. [9, 10].

Primary Health Care (PHC), established to redress disparities in healthcare accessibility in India, has encountered obstacles in fulfilling its goals, especially in rural areas due to inadequate staffing and limited resources. Inadequate monitoring, limited supplies, low community engagement, and subpar care provision underscore the need for a potential alternative and integrated care model. [11, 12].

Considering that enhancing child health and ensuring healthcare for all is a fundamental objective of sustainable development, it is imperative to tackle these concerns underscored in the Astana Declaration of 2018. [13, 14].

WHA Rehabilitation 2030 initiative emphasizes the global need for rehabilitation services, particularly in low- and middle-income countries, and promotes the development of essential frameworks and interventions, this calls in for a enhanced rehabilitation services in low- and middle-income countries like India. [15].

The fragmented nature of the healthcare system and the unequal distribution of healthcare facilities, coupled with limited specialist care, have prompted concerns regarding the need to establish robust and specialized outreach centers that ensure maximum accessibility and utility. [6].

To enhance the utility of outreach clinics, it is advised to establish customized outreach centers tailored to the specific needs of the community, preferably located in schools. School-based healthcare services have been shown to enhance accessibility and utilization, involving various healthcare professionals such as physicians, nurses, physical therapists, occupational therapist, speech language pathologists, community workers, and even teachers. [6].

Previous research has focused on estimating the prevalence of disabilities among preschool children in Western regions, leaving a significant data gap on the challenges faced by caregivers and disabled preterm children in Tamil Nadu. This lack of evidence hampers the development of policies, model outreach clinics, and support systems for parents and guardians of preterm children with disabilities, potentially leading to preventable long-term complications due to delayed access to healthcare and screening.

Given the increasing rate of NDD in early childhood and lack of accessibility it is imperative to regularly screen children for potential risk factors and provide timely interventions to prevent long-term complications particularly in rural areas with limited access to specialist care.

A two-step approach involving screening and definitive testing at healthcare facilities, along with standardized assessments for early identification of developmental abnormalities, is crucial for improving outcomes in children with NDD.

SRM Institute of Science and Technology (SRMIST) Kattankulathur, in alignment with the developmental goals of Unnat Bharat Abhiyan (UBA) has launched UBA-SRMIST in 2019 under the guidance of the University Grants Commission/ Ministry of Human Resource and Development/ All India Council for Technical education. UBA-SRMIST has adopted twelve rural villages in Kancheepuram and Chengalpattu and has conducted several welfare programs including awareness camps, screening programs and providing COVID-19 vaccination.

This study aims to evaluate the developmental outcomes of preschool children in twelve adopted villages of UBA-SRMIST, Kattankulathur, and provide early intervention programs by establishing a specialist outreach/model clinic which will help to uplift the village and generate data that can inform policies and interventions aimed at supporting parents, enhancing accessibility, and reducing the  burden of caregiver.

## Primary objectives

To assess the prevalence of neurodevelopment disorder among children in the UBA adopted villages of SRMIST.

To develop a model physiotherapy centre and assess its impact on reducing the burden among the caregivers of children with neurodevelopmental disorders in rural areas of Tamil Nadu.

## Materials and methods

### Participants

A single arm study will be conducted among children aged 3 to 5 years attending Anganwadi centers in SRM UBA adopted villages who will be screened for neurodevelopmental disorders with parental consent. Those identified with NDD or previously diagnosed with any form of NDD will be included in the study.

## Procedures

Given the need for a comprehensive understanding of the local community to plan a specialist outreach clinic effectively, this study will be conducted in phases to assess prevalence, healthcare needs, and resource availability. This is crucial for establishing a specialized outreach clinic in a village. [16].

The initial phase of the research will entail the gathering of fundamental data from the twelve villages adopted by SRMIST namely, Kolathur, Chettipuniyam, Orathur, Nattarsanpattu, Anjur, Thenmelpakkam, Pattaravakkam, Kalivanthapattu, Baburayanpettai, Minnal Chithamur, Kilathivakkam and Chunambedu.

The fundamental details will include information on the number of panchayats (basic governing institution in Indian Villages), anganwadis(Rural child care centre in India), and the demographics of preschool children registered in Anganwadi.

Subsequent to the fundamental data collection and establishment of a rapport with the Panchayat authorities and Anganwadi personnel, a preliminary screening will be conducted by physiotherapists on all Anganwadi children aged between 3 to 5 years in 12 SRM UBA adopted villages after obtaining the parental consent. This screening aims to comprehensively enumerate data for determining the prevalence of Neurodevelopmental Disorders (NDD) among these children. The rapport established with village authorities will help in gaining confidence of common people and will help in improving their adherence.

Physiotherapists, in collaboration with parents, will complete a screening checklist. This checklist will primarily focus on detecting developmental delays, DCD, autism, and ADHD, as these are the most commonly observed NDDs among preschoolers. Information on children previously diagnosed with NDD and their history of treatment/rehabilitation will be gathered retrospectively.

Upon identifying the prevalence of neurodevelopmental disabilities, model centers will be established in villages with high prevalence rates. Consideration will be given to ensuring that these centers are accessible to the entire population, including nearby villages by discussing with the village heads. The establishment of model clinics will be based on the identified needs. While the initial plan is to set up one model center, if the prevalence is high and there is a demand, multiple model centers will be established by clustering the villages based on NDD distribution.

The model center, or specialist outreach center, is envisioned to have trained physiotherapists for children's rehabilitation, with pediatric physicians visiting the centers for progress checks and further evaluations. Equipped with essential tools such as swiss ball, peanut ball, obstacle for gait training, balance boards,etc., the center will provide pediatric rehabilitation and group therapies for children. Discussions and arrangements will be initiated with village authorities to position the model center close to or within school/Anganwadi premises.

Subsequent to NDD identification in children, baseline assessments of NDD will be conducted alongside qualitative assessment of caregivers' perspectives and experiences regarding accessing rehabilitation services in the community. Quantitatively, caregiver burden will be evaluated using a caregiver burden scale.

Upon enrollment, children will undergo physiotherapy rehabilitation for one year under a trained physiotherapist, with routine screenings by pediatricians. The neurodevelopmental rehabilitation will include sensorimotor training, motor learning, sensory integration, motor training and neurodevelopmental therapy which will be tailored according to the individual’s needs and the severity and type of disorder. Treatment will be given in person, in group or as individual therapy. The entire timeline and flow have been illustrated in Table 1.

**Table 1:** Spirit timeline for the study

	Enrolment and allocation		Post allocation				Endline evaluation	Close-out
Time point	− t1	t0	t1	t2	t3	t4	t5	t7
Preliminary screenings	X							
Parental consent	X							
Analysis of prevalence	X							
Eligibility screen		X						
Obtained signed parental consent		X						
Allocation		X						
Baseline assessments		X						
Establish model centre		X						
One year rehabilitation			X	X	X	X		
Routine follow up screenings by pediatricians			X	X	X	X		
Baseline tools	X	X					X	
Effectiveness of intervention		X					X	
Caregiver burden		X					X	
Data entry and analysis	X						X	
Share findings with authorities	X							X
Final reports								X
Plan future studies								x

Each t indicates 3 months of time span

Treatment dose, intensity and sessions will be modified based on functional goals. After the year-long intervention, children and caregivers will undergo an end-line evaluation to gauge the effectiveness of the specialist outreach/model clinic in improving children with NDD and alleviating caregiver burden which will be assessed by the Physiotherapist who provided rehabilitation.

## Outcome measures

### Primary outcome measures

#### Caregiver Burden Scale

The Caregiver Burden Scale is a measurement tool developed by Vadivelan K et al., through empirical analysis involving caregivers of children with disabilities in Tamil Nadu, considering sociocultural factors. It comprises 15 items rated on a 5-point Likert scale, ranging from 'strongly disagree' (1) to ' strongly agree' (5) [5]. This scale has demonstrated strong internal consistency, with a Cronbach's alpha exceeding 0.8. [17]. The higher the burden, lesser the quality of life.

## Outcome measures for preliminary screening

### Little Developmental Coordination Disorder Questionnaire (Little DCDQ)

Little Developmental Coordination Disorder Questionnaire (Little DCDQ) is a parental assessment tool designed to screen children aged 3 to 5 for developmental coordination disorder. It exhibits good validity (0.73–0.87) and shows high agreement with the Movement Assessment Battery for Children – 2 (MABC-2). Little DCDQ comprises 3 domains, each containing 5 items, assessing fine motor skills, execution control, and overall coordination. Responses are rated on a 5-point Likert scale, with specified cutoff values for DCD classification and screening. [18].

## Ages and Stages – 3 (ASQ—3)

ASQ 3 developed by Squires and D. Bricker, is a parent-administered screening tool with five domains covering gross motor skills, fine motor skills, communication, personal-social interactions, and problem-solving abilities. ASQ—3 is strongly endorsed by the American Academy of Pediatrics due to its robust psychometric properties and user-friendliness. It demonstrates high sensitivity and specificity in identifying developmental delays in children aged 2 to 60 months and shows good agreement with DASII and Bayley scales.[19, 20].

## ADHD rating scale IV – preschool version

The ADHD Rating Scale IV – Preschool Version is a reliable tool for detecting ADHD in children aged 3 to 5 years. Parent rated version had established an excellent test–retest reliability (0.85–0.95) and an internal consistency of 0.95. Scale consists of 18 items rated on 4-point likert scale (0-never/rarely to 3 – very often) [21].

## Indian Scale for Assessment of Autism (ISAA)

ISAA was created by the National Institute for the Mentally Handicapped to diagnose and assess the severity of autism. The scale has 40 items under 6 domains scored based on 5-point likert scale. The interpretation of scores are: no autism (score < 70), mild autism (scores between 70–160), 107–153 (Moderate autism) and severe autism (> 153). [22]. It demonstrates reasonable sensitivity in screening for autism in children aged 2–5 years. [23].

## Statistical analysis

The collected data will be entered into a database and analyzed using SPSS. Baseline demographics including participant details, obstetric history, educational status, family history and other relevant details will be visualized using Tableau. Baseline characteristics will be summarized as frequencies and percentages for categorical variables and as means ± standard deviations (or medians with interquartile ranges for non-normally distributed data) for continuous variables. Inferential statistics: Chi-square test: We will use this to analyze associations between categorical variables, such as caregiver burden severity and prior diagnosis of neurodevelopmental disorders (NDD). t-test: Applied to compare means between two groups, such as pre- and post-intervention caregiver burden scores.

ANOVA: Used to compare differences in continuous outcomes, such as caregiver burden scores and child developmental outcomes, across multiple groups (e.g., different clinics or geographic regions).

Regression analysis: Binary Logistic Regression: This will help us identify the impact of the intervention on the binary outcome (reduction in caregiver burden, yes/no), controlling for potential confounding variables (e.g., socioeconomic status, severity of NDD).

Visualization tools: Tableau will be used to create dashboards summarizing key indicators, such as clinic utilization, caregiver burden trends, and regional prevalence rates.

## Qualitative analysis

Parents of the children who received rehabilitation in the model clinic will be interviewed face to face to understand the impact of model outreach clinic in reducing their burden and improving the access and child care. The entire interview will be audio recorded with parental consent and will be analyzed using grounded theory analysis following which themes and subthemes will be derived.

## Discussion

The anticipated primary outcome of the research will focus on the influence of the model center on reducing the burden among caregivers of children with NDD in the adopted villages affiliated with SRM Institute of Science and Technology, situated in Kattankulathur. Moreover, alongside the dissemination of the research findings, it will facilitate the strategic development of specialized services and the provision of care, as well as the appropriate referral of patients to higher levels of healthcare as necessary.

The Indian government has initiated UBA Scheme for the upliftment of rural villages. The primary objective of the UBA scheme is to engage higher educational institutions in research and understanding the realities of these communities at grass root level to contribute to the development of the village.

SRMIST under UBA has signed a Memorandum of Understanding with the Tamil Nadu State Institute of Rural Development and Panchayat Raj (SIRD&PR). The aim of the MoU was aligned with nine Local Sustainable Developmental Goals (LSDG) which included healthy village and child friendly village.

This research tries to fulfil these objectives by involving health care professionals in village level care via which both hospitals will be benefited by understanding the realities and also the village will be uplifted by reducing disability and caregiver burden. Also, the establishment of local outreach centre will help reducing the time and travel burden of parents making sure that the child receives treatment to the maximum.

The findings of this investigation will yield essential data concerning the frequency of developmental irregularities and disabilities among preschool children in twelve adopted villages linked with SRM, located in Kattankulathur. This data has the potential to steer the formulation of suitable strategies and protocols for the early implementation of rehabilitative measures, thereby alleviating the burden of disability among preschool children.

Furthermore, this study can act as a blueprint for establishing similar centers in various regions within Tamil Nadu. The data gathered will serve as a compass for governmental bodies and healthcare agencies in devising pertinent strategies and protocols to mitigate the prevalence of disability among children in Tamil Nadu.

The effectiveness of specialized outreach clinics in rural locations has been established in improving patient outcomes by avoiding the requirement for referrals beyond the community (out-of-community care) and strengthening the continuity of care. Among the various frameworks available for delivering specialized care, outreach specialist clinics have exhibited scalability. [16, 24].

The establishment of a specialized outreach program for pediatric rehabilitation aims not only to enhance access but also to ensure the equitable dispersion of healthcare services and communal involvement. This initiative will not only bolster community engagement but also enhance awareness and motivation among neighboring villages and communities to actively partake in rehabilitation efforts by dispelling the associated stigma. [24].

The outcomes of this study strive to fulfill the exigency for substantiating the efficacy of outreach clinics in rural and underprivileged areas despite previous studies highlighting the dearth of robust research evidence in this domain. [16].

## Supplementary Information

Supplementary Material 1.

Supplementary Material 2.

## Data Availability

No datasets were generated or analysed during the current study.

## Abbreviations

UBA: Unnat Bharat Abhiyan
SRMIST: SRM institute of science and technology
NDD: Neurodevelopmental disorder
ADHD: Attention deficit hyperactivity disorder
DCD: Developmental coordination disorder
ASQ: Ages and stages questionnaire
NFHS: National family health survey
